# Wisconsin Medical Act

**Published:** 1879-07

**Authors:** 


### The Wisconsin Medical Act.

We have received a copy of the act, as read the third time in the Senate:—

SECT. 1. A person practicing in the State must have a diploma, or pass an examination before the State Board of Examiners.

SEC. 2. The Governor shall appoint a board of examiners, of six physicians (four regular and two homœopathic), and those shall appoint a seventh.

SEC. 3. Defines the organization of the Board.

SEC. 4. The Board shall examine diplomas and candidates. Holders of genuine diplomas shall pay $2.00; of spurious diplomas, $20.00.

SEC. 5. Persons not holding diplomas may be entitled to practice after examination.

SEC. 6. Every such license shall be recorded within thirty days.

SEC. 7. The Register of Deeds shall keep the records.

SEC. 8. Fee for candidates for examination to be $5.00.

SEC. 9. Payment of Examining Board.

SEC. 10. Examinations defined.

SEC. 11. Dishonorable conduct may forfeit certificates.

SEC. 12. Defines a practitioner of medicine.

SEC. 13. If any physician practicing medicine in this State shall write, or cause to have printed upon any prescription, the words, "No Duplicate," any druggist, apothecary, or vender of medicines who shall duplicate a prescription so written or printed upon, without the consent of the physician writing the prescription, shall, on conviction thereof, be subject to a fine of ten dollars ($10) for each and every offence, together with the costs of suit.

SEC. 14. Itinerant venders of drugs to pay $100 per month.

SEC. 15. Penalties for disregarding this act.

The remaining sections are formal; the act to take effect after August 15th next.—*Medical and Surgical Reporter.*

